# Enhancing Gender

**DOI:** 10.1007/s11673-021-10163-7

**Published:** 2022-02-07

**Authors:** Hazem Zohny, Brian D. Earp, Julian Savulescu

**Affiliations:** Oxford Uehiro Centre for Practical Ethics, University of Oxford, 16-17 Saint Ebbe’s St, Oxford, OX1 1PT UK

**Keywords:** Transgender, Well-being, Enhancement

## Abstract

Transgender healthcare faces a dilemma. On the one hand, access to certain medical interventions, including hormone treatments or surgeries, where desired, may be beneficial or even vital for some gender dysphoric trans people. But on the other hand, access to medical interventions typically requires a diagnosis, which, in turn, seems to imply the existence of a pathological state—something that many transgender people reject as a false and stigmatizing characterization of their experience or identity. In this paper we argue that developments from the human enhancement debate can help clarify or resolve some of the conceptual and ethical entanglements arising from the apparent conflict between seeking medicine while not necessarily suffering from a pathology or disorder. Specifically, we focus on the welfarist account of human enhancement and argue it can provide a useful conceptual framework for thinking about some of the more contentious disagreements about access to transgender healthcare services.

## Introduction

In recent years, there has been growing awareness of the social and political needs of transgender (trans) people: to correct injustice, prevent mistreatment, and make the world safer for their flourishing. Terminology in this area is contentious (Bettcher 2020). However, a common understanding, including within the relevant communities, is that a trans person is someone whose deeply-held sense of themselves as a boy/man, girl/woman, or non-binary person (among other potential gender self-identifications) does not correspond to their birth-designated sex category in a way prescribed by the dominant culture. According to the prevailing gender ideology of many cultures, the normatively appropriate gender role for a person with male-typical bodily features, such as a penis, is boy/man (characterized by traits and behaviours considered masculine within the culture) whereas the normatively appropriate gender role for a person with female-typical features, such as a vulva, is girl/woman (characterized by traits and behaviours considered feminine within the culture). Often, having a sense of oneself as more appropriately belonging to a sex or gender category other than the one that traditionally corresponds to one’s sex-typed features is accompanied by significant distress. This distress may have multiple causes, including biological, psychological, and social factors (such as teasing or bullying rooted in social stigma) and often involves a strong and persistent discomfort with, or alienation from, one’s sexual anatomy and/or culturally prescribed gender role (Cooper et al. 2020). In a medical context, assuming that certain conditions are met, this distress and discomfort is termed “gender dysphoria.”

However, within the medical sphere, the desire to improve care pathways for trans people—in particular those who are dealing with gender dysphoria—has faced a dilemma. On the one hand, access to certain medical technologies, including hormone treatments or surgeries, where desired, may be beneficial or even vital for some gender dysphoric trans people. But on the other hand, access to medical technologies typically requires a diagnosis, which, in turn, seems to imply the existence of a pathological state—something that many transgender people reject as a false and stigmatizing characterization of their experience (or identity). In particular, they reject the idea that “being transgender” either is, or is necessarily indicative of, a disorder of any kind (Burke 2011).

Linked to this dilemma are three further controversies. The first relates to the possibility of co-occurrent psychological differences or difficulties: while being transgender is not a disorder of any kind, *in some cases* gender dysphoria may be indicative of other disorders—disorders that foster the experience of gender dysphoria or that may reduce the likelihood that the use of medical technologies to try to alleviate the dysphoria directly will succeed. For instance, some researchers have found a higher prevalence of autistic traits among trans individuals compared to the general population, especially among those designated female at birth (de Vries et al. 2010; Stagg and Vincent 2019). The presence of autistic traits does not of course entail that one is not transgender or that having or adopting a trans identity is not among the most coherent options for making sense of one’s sexed embodiment and associated experiences in a given social context. Moreover, many people contest the idea that autistic traits should, themselves, be pathologized as “disordered” as opposed to embraced as part of human neurodiversity (Kapp et al. 2013). Nevertheless, the presence of such traits or (other) signs of neuropsychological difference may suggest that an alternative or broader range of medical and/or non-medical interventions might productively be explored as a way of alleviating the felt dysphoria, apart from, or possibly in addition to, e.g., interventions to alter one’s sexual anatomy.

Second, in some cases the unease or distress of gender dysphoria may arise largely due to external factors rather than internal ones. For instance, the distress experienced by some trans individuals may be due, in large part, to society’s response to their unconventional gender presentation or expression. Diagnoses are typically theorized as evaluations of problems internal to a person’s body or mind, and some have argued that a diagnostic approach (so conceived) undervalues the role of external factors or dismisses this potential relationship between internal distress and societal discrimination (Lev 2004; Schulz 2018).

Finally, not all trans people suffer from gender dysphoria or experience distressing levels of social discrimination. But these individuals may nevertheless desire to alter various sex characteristics for purposes of identity exploration, self-affirmation, or what is sometimes called creative transfiguration (Ashley and Ells 2018). In other words, rather than trying to “treat” gender dysphoria (eliminate a negative state), they may seek to “enhance” the form or function of their embodiment in keeping with their identity and values. The current criteria for accessing medical technologies for purposes of gender affirmation (i.e., changing one’s physical embodiment so as to better accommodate one’s deeply-held sense of oneself as a gendered being in a given society) overlooks this group entirely.

In this paper we argue that developments from the debate on human enhancement can help clarify and potentially resolve some of the conceptual and ethical disagreements arising from the issues just noted. Specifically, we will focus on the welfarist account of human enhancement, which characterizes enhancement as “any change in the biology or psychology of a person which increases the chances of leading a good life in the relevant set of circumstances” (Savulescu, Sandberg, and Kahane 2011, 7). Our aim is to show how this account can help sidestep the seeming tension between diagnosis and pathology: that is, the tension between needing or desiring access to a medical technology without (necessarily) having a relevant disease or disorder. It should also help us in deliberating about cases where (1) a psychological disorder co-exists with a desire to access gender-affirming technology, (2) the distress experienced by a gender dysphoric trans person is primarily due to external factors, and (3) where there is little or no distress at all.

Our argument unfolds in four parts. First we elaborate on the challenges facing what we will call the “diagnostic model” and its criteria for accessing transgender healthcare services like hormone treatments or surgeries. We then introduce the welfarist account of enhancement and the general framework it provides for characterizing the goals of medicine, before proceeding to elaborate on what this means in the context of gender affirmation. Finally, we explore how it can help clarify thinking about more complex cases of individuals seeking to access gender affirmation services. We will not touch on instances of young children or other insufficiently autonomous decision-makers accessing these services, which raise more specific ethical challenges, though we hope some of our conclusions here may be relevant to articulating and thinking about some of those ethical challenges more clearly.

## The Diagnostic Model

The approach of the diagnostic model to gender affirmation healthcare has come under increased fire in recent years (Schulz 2018; Riggs et al. 2019; Lipshie-Williams 2020; Spanos et al. 2021). On this approach, those who seek access to gender affirming services must be granted approval by a mental health practitioner on the basis of meeting the criteria for a diagnosis of gender dysphoria or gender incongruence. (The former term is used in the fifth Diagnostic and Statistical Manual of Mental Disorders and the latter is used in the International Statistical Classification of Diseases and Related Health Problems.)

These diagnoses entail marked, prolonged unease or distress related to a perceived “mismatch” between one’s gender identity and their birth-categorized sex based on genital appearance. It is evidenced by a strong desire for a gender presentation that better accords with their felt gender and is associated with clinically significant distress or impairment in daily life (Zucker, Lawrence, and Kreukels 2016).

The rationale for the diagnostic model is straightforward. Diagnoses are—typically and appropriately—indispensable for accessing medical care. Given existing models of healthcare access, which require a medically valid diagnosis, simply doing away with the diagnostic category of gender dysphoria would effectively close the door on those trans people who need or desire access to gender-affirming medical technologies. Also, insurance companies and public health systems typically require a diagnosis to justify a medical intervention before covering it. Getting rid of the diagnostic model would, under current conditions, prevent trans people from being covered.

We have already touched on the controversies associated with this model. The first relates to “pathologization” which refers, roughly, to the process of conceptualizing something as a disease state that plausibly ought not to be conceived of that way (e.g., because it is a “normal part of life”).^1^ The requirement of seeing a mental health professional to access gender affirming services has been described as unnecessarily pathologizing and stigmatizing (Bockting et al. 2004). Instead, trans people generally wish to access such services without the implication of potential mental illness and its associated stigma. Both the DSM-5 and ICD have moved away from using the term “disorder” in their diagnostic labels for trans people and have relocated these entries to sections in their manuals unrelated to sexual dysfunction and paraphilic disorders. The goal was explicitly to reduce stigma toward trans people. On the other hand, even without the term “disorder,” receiving a medical diagnosis still implies the presence of a pathology of some kind, or at least a medicalized or quasi-medicalized “condition” with “symptoms,” which in turn can justify the use of medical treatment.

Moreover, there is a concern that the requirement of significant unease for this diagnosis may encourage trans individuals to embrace a “distress narrative” in order to minimize the barriers to accessing needed medical technologies—indeed, this has been a long standing concern in the trans literature (Sandy Stone 1987), with some evidence that this requirement incentivizes trans individuals to rehearse whatever is likely to grant them the quickest access these services (Davy 2010; Hines 2007).

Another, perhaps subtler, problem relates to how diagnostics is typically framed in terms of uncovering “abnormal” functioning as a means to then restoring normal functioning (Brinkmann 2016). This framing tends towards binary thinking in terms of gender (Inch 2016). If normal functioning entails an “alignment” between one’s male or female biology (i.e., sex-typed features) on the one hand, and one’s gender identity on the other, it follows that “normal functioning” can be restored by altering elements of one’s biology to bring it into line with how one identifies. This then leads to the diagnostic criterion of strongly identifying with either a male or female gender in order to qualify for gender affirming services, reinforcing the binary. Moreover, these services are often set up with a one-size-fits-all assumption about “normal functioning” when it comes to gender, with the normative end goal of “full” medical transition (Dembroff 2019).

But this has a number of negative consequences. Firstly, it may encourage transgender individuals who do not necessarily fully or exclusively identify with one particular sex or gender category to nevertheless cast themselves within the stereotypical binary in order to access some of these services (Hines 2007; Bettcher 2014). More than that, it might even colour the self-perception of some transgender individuals, encouraging those ambivalent about their gender, or who might otherwise reject the idea of fitting squarely within one binary category or the other, to instead think that they must really be *either* a boy/man *or* a girl/woman. But one may experience dysphoria with their current state without necessarily seeking to “fully” affirm or transition to any one particular gender. Indeed more and more trans individuals are electing what have been called “transgender-ish interventions,” wishing only for the administration of so-called cross-sex hormones (e.g., testosterone for those with female-typical anatomy; oestrogen for those with male-typical anatomy) without any surgery, and in the case of transgender men some may wish to have mastectomies without pursuing phalloplasty, and so on (Cocchetti et al. 2020).

These grey areas become further complicated when those seeking access to gender affirming technologies do in fact suffer from psychological burdens that may appropriately be characterized as disorders under the diagnostic approach. As noted, there is much controversy about the apparently higher prevalence of autistic traits among trans individuals compared to the general population (de Vries et al. 2010; Stagg and Vincent 2019), with reports that these individuals are blocked from accessing gender affirming healthcare on that basis (White 2016). Guiding these decisions to block access is presumably a belief that, due to the presence of some putative psychological disorder, transitioning will not actually mitigate the distress they experience. It may even be believed that the existence of a different diagnosable psychological condition suggests that the desire to transition is not in some sense “authentic.” Instead, the desire might be seen as symptomatic of an “underlying” condition. The diagnostic model provides a limited framework for thinking about these cases, even as there is growing agreement to depathologize the experience of gender dysphoria (and in some corners, as mentioned, also autism among other conditions). In other words, the diagnostic model tends to attribute the cause of gender dysphoria to any psychological disturbance, which then becomes the primary object of “treatment.”

There may also be concerns about trans individuals in communities that are prejudiced against their gender presentation. These individuals may feel pressured to transition not because of a deep-rooted sense of dysphoria stemming from stable psychological traits vis-à-vis sexual anatomy but to reduce the stigma against their appearance or behaviour. Or, more complicatedly, it may be that a significant cause of their sense of dysphoria is others’ negative reactions to their gendered presentation or sense of self.

The diagnostic model is not well-suited for accounting for this form of distress. The goals of medicine are typically understood as treating conditions conceived of as “internal” to a patient, rather than distress arising from external circumstances, like one’s cultural surroundings. For instance, many would find it abhorrent if individuals were allowed to access medical interventions to alter the colour of their skin to avoid racist discrimination.^2^ That, some might argue, would be a misuse of medical interventions: surely we should combat prejudice and discrimination rather than try to side-step it in such a manner by changing the individual. Yet there is evidence that in some countries, such as in Iran, where homosexual acts are punishable by death but where there is tolerance to the idea of being trans, gay men and women are indeed pushed to undergo gender reassignment surgery to avert homophobic discrimination (Ali Hamedani 2014).

And lastly, not all trans people necessarily experience gender dysphoria or incongruence between their gender identity and body but may wish to access some gender affirming services nonetheless. In these cases, the idea would not be to ameliorate distress (although the individual might believe the intervention(s) will make them happier). Instead, the person seeking gender-affirming interventions into their biology may be driven by a belief that this will make their lives more meaningful or valuable; they may even have aesthetic or experimental reasons to pursue such interventions (sometimes called “creative transfiguration” in the trans literature (Ashley and Ells 2018)). Rightly or wrongly, the diagnostic model makes no allowance for these individuals (Dembroff 2019).

To summarize, the diagnostic model raises questions related to 1) pathologizing trans people (even if implicitly), 2) cases where a psychological difference or disorder co-exists with a desire to medically affirm a gender identity, 3) how to think about internal versus external distress, and 4) what to make of trans or non-binary individuals who may not be severely distressed but who nevertheless might wish to access at least some gender-affirming services.

In recent years, some transgender advocates have offered an alternative to the diagnostic model which they suggest avoids some of the above sorts of problems. This alternative has been called the “informed consent” approach, and it involves allowing trans individuals to access gender affirming services insofar as they themselves reasonably deem them advantageous and they give their free and informed consent. On this approach, a special mental health evaluation—along with any associated diagnosis—is not seen as a requirement for accessing the services (Schulz 2018), though the healthcare provider, in keeping with their professional code of ethics, must still make a determination that the “treatment” is in the best interests of the individual and that the consent given is ethically valid.

Seemingly, insofar as one endorses this approach, one may at least partially side-step the ethical controversies we have identified. However, rather than side-stepping those controversies, our aim is to provide a conceptual framework that can help us navigate them somewhat more clearly. This framework may also be seen as philosophical defence of at least some of the conceptual and normative presuppositions of the informed consent model. We will describe and explain this framework in the following section.

## The Welfarist Account of Enhancement

The welfarist account of human enhancement, as we noted, characterizes enhancement as:

“Any change in the biology or psychology of a person which increases the chances of leading a good life in the relevant set of circumstances” (Savulescu, Sandberg, and Kahane 2011, 7).

This definition was developed to help clarify ethical disagreements about enhancement as well as conceptual disagreements related to the treatment-enhancement distinction. This distinction relates to the idea that treatments intervene in the body or mind to restore or maintain normal bodily and mental functioning, while enhancements augment functioning in ways that go beyond mere restoration or maintenance (Juengst 1998; Daniels 2000). Commentators on this debate often question whether a clear distinction can be non-arbitrarily drawn, but much of this debate boils down to disagreements about what the proper goals of medicine are and whether we can scientifically identify what is “normal.” While traditionally medicine aims at restoring or maintaining normal functioning, enhancements put pressure on a) whether medicine is in fact limited in this way (there is a long history of medical practitioners using their expertise and associated technologies far more expansively than in this restricted sense (Boorse 2016)) and b) even if we can agree on what normal functioning is, it is unclear what is morally special about it and why we should restrict medicine’s scope to promoting it alone.

Taking a cue from these two points, the welfarist account effectively abandons the treatment-enhancement distinction. After all, it is strange to think that treatments are valued merely because they restore or help retain normal functioning. We value normal functioning—to the extent that we agree on what it is—only to the degree that it enables us to do things we value and ultimately to the degree that it promotes our ability to have good, worthwhile lives. A treatment is valued as a treatment to the extent that it will likely make a patient’s life go better. Similarly, if an intervention radically augments functioning beyond the normal, if that augmentation is to be valuable to us it will only be so to the extent that it likely makes our lives go better. Indeed, an intervention may *reduce* our functioning but still be valuable (Earp et al. 2014a). For instance, vasectomies hinder “normal” functioning yet are considered standard medical procedures that may be highly valuable in certain circumstances, given the patient’s personal and/or relational needs and values. More radically, some may wish to hinder their ability to recall, or at least feel the sting of, certain events or memories if that mitigates the impact of past trauma on their lives (Brunet et al. 2018). It is that prudential value—that is, the thing about an intervention that makes it good *for* the person undergoing it—that unites all these interventions. And it is what, on a welfarist account, makes them all enhancements.

The abandonment of the treatment-enhancement distinction is reflected in the account’s inclusion of *any change* in the biology or psychology of a person*.* It is irrelevant whether that change is to repair some bodily or mental dysfunction (what might normally be considered “treatment”), to augment normal functioning (what is normally understood by “enhancement”), or even to intentionally diminish certain biological or psychological capacities. All such changes *could* count as enhancements on the welfarist definition, so long as they expectably increase the well-being of the person undergoing the change.

On this view, then, a medical treatment is a subclass of enhancement—it is one way to expectably increase well-being by changing the body or mind, since “treating” a disease or disorder typically has that effect. But there is no special moral significance to addressing a disease, disorder, dysfunction, or any other (typically negative, aberrant) condition normally seen as an appropriate target of “medicine.” What matters is the increase in well-being, relative to some suboptimal state, all else being equal.

A corollary of this account is the welfarist account of disability:

“Any state of a person’s biology or psychology which decreases the chance of leading a good life in the relevant set of circumstances” (Savulescu, Sandberg, and Kahane 2011, 7)*.*

Here too, the point is not to identify an “objective” dysfunction or pathology by virtue of which the person is disabled but rather to highlight any biological or psychological attribute that counts against the person’s well-being in the given set of circumstances. Of course, changing the *circumstances* may often be the most appropriate course of action when well-being is at stake (or some combination of the circumstances and the biology or psychology of the person), but we will come on to that below.

In shifting the perceived purpose of medical interventions from the restoration or maintenance of normal functioning to the improvement of well-being, the welfarist account reconceptualizes the goals of medicine are. It ties in with a long-standing argument that medicine is ultimately about, and *ought* to be about, patient benefit and not the restoration or maintenance of normal functioning (Hesslow 1993). It expands the purpose of medicine from being only about combating pathology. Medicine, rather than being fundamentally or exclusively concerned with identifying diseases and curing them, becomes about using science to identify which means of promoting well-being via changes to the body and mind are most likely to be effective and safe, as well as broadly ethically acceptable (vis-à-vis all relevant moral principles, such as justice, for example, or autonomy) (Foddy, Kahane, and Savulescu 2013).

Thinking of medicine this way retains the usefulness of diagnostics: a diagnosis helps guide what sorts of interventions are likely to improve a patient’s well-being. But it disconnects the conceptual link between receiving a diagnosis and being in some sense dysfunctional. This approach has been applied in the context of psychiatry and diagnosing mental illnesses in general (Roache and Savulescu 2018) but not specifically to gender dysphoria and more broadly the desire of some individuals to access gender affirming interventions. To come back to the topic at hand, the welfarist account turns the question of whether gender dysphoria is a disorder or not into a moot point for the purposes of accessing gender affirming healthcare.

We see this approach as being broadly in line with what the informed consent model advocates. However, the informed consent model is not designed to give us the conceptual framework for thinking about what these healthcare services are for, or how to cash out the—at times competing—values at stake when deliberating about who to provide them to and who ought to pay for them. More than that, it does not provide a framework for thinking about the cases discussed in the remaining sections. We note, however, that we do not propose a welfarist approach *in place* of the informed consent model, but rather, we see it as a complementary conceptual toolkit that may help fortify it against objections that it is fundamentally inconsistent with the proper goals of medicine.

## Well-Being and Gender Dysphoria

Broadly, the framework that emerges from the welfarist account for individuals with gender dysphoria is this: a sense of incongruence between one’s gender identity and one’s birth-categorized sex can reduce one’s chances of leading a good life. There may be many reasons for this incongruence, and its impact on well-being may in large part be determined by one’s circumstances (whether personal or social). For the welfarist account, that incongruence is disabling to the extent that it tends to reduce how well a life can go. However, the term disabling here is not meant in a traditional, medical sense—rather, it is intended to be used synonymously with disadvantageous. That is, the unease or distress associated with the sense of incongruence is disadvantageous from a welfare perspective (see Zohny 2016 for the welfarist account’s scope for what counts as disability but also Kahane and Savulescu 2009 for a competing view).

In contrast, to undergo a change in one’s biology (and/or psychology) that neutralizes or diminishes that unease or distress could be considered an enhancement. We use “could” here, rather than “is” or “should be,” because whether some change is or is not an enhancement will depend on the likelihood that it will on balance improve the chances of the affected person leading a better life in their particular circumstances. For instance, taking certain hormones to change one’s gendered embodiment may be associated with side-effects and risks (Getahun et al. 2018). These would be disabling to the extent that their use is likely to diminish an individual’s well-being. However, if the gain in well-being from the diminished dysphoria that is reasonably expected to arise from the hormones outweighs their disabling or disadvantaging impact, that would make them (all things considered) enhancing.

It is also possible, in principle, that a change in the psychology of a person with dysphoria, without associated changes in biology, could be enhancing for some individuals (Lemma 2018, 2020; D’Angelo 2020). For instance, it may be that in some cases, talking therapy alone could help to relieve distress or foster a sense of acceptance about one’s body and how it relates to one’s gender identity in a given social context. In some cases this may be preferable to biological interventions if, for instance, talking therapy is likely to be effective and the individual in question has a pre-existing physiological condition that makes the biological interventions excessively risky.

In some cases, neither of these pathways for intervention may be viable options due to the circumstances of the individual in question (for instance, they may reside in a country that provides no care at all for trans people). The welfarist account also asks us to take into account what could be changed about their circumstances to mitigate their distress.

The central question on the welfarist approach, then, is how to identify which (bio, psycho, and/or social) intervention(s) will be likeliest to promote a particular trans individual’s overall well-being in their particular (social, legal, relational, financial, medical) circumstances. *That* is the driving question and *not* whether this individual “actually” has a disorder (as though the question could be objectively decided, that is, without appeal to potentially disputable value judgments) or whether what they seek conforms with “normal” functioning (however defined). Gender dysphoria may be a diagnosis for some individuals seeking to access gender affirming healthcare; but whether that diagnosis indicates the presence of a pathology is irrelevant for medical decision-making. Instead, what matters in terms of the goals of medicine and of transgender healthcare services is identifying an ethically permissible way to best improve the well-being of a trans person, given the available (including, but not necessarily limited to, medical) tools and their particular circumstances.

In other words, the welfarist account suggests there is a prima facie reason to promote access to transgender healthcare services—whether biological, psychosocial, or both—when doing so will likely improve the well-being of the person gaining that access, compared to alternatives. In fact, on this account there is a prima facie reason to allow access to these services even if one is not distressed about their sex-typed bodily features but nevertheless wishes to alter their gender presentation for other reasons, such as artistic or experimental ones.

After all, in many jurisdictions adults need not provide a strong justification, let alone a pathology-based justification, for why they desire to undergo even highly intrusive and relatively risky procedures—including so-called “cosmetic” genital surgeries—so long as they believe it will leave them better off (Shahvisi and Earp 2019). This is not to suggest that such cosmetic surgeries (or associated marketing practices or background cultural norms driving the demand for them) are beyond critique (Braun 2010; Boddy 2016), nor that cosmetic surgeries and trans surgeries are fully analogous (see Latham (2013) and Heyes and Latham (2018) for careful discussions about the potential disanalogies). Instead, this is merely to note that if competent adults should be allowed to have their anatomy changed for “merely” cosmetic reasons—without undergoing a special mental health evaluation or receiving any kind of diagnosis—it is hard to see why a sufficiently autonomous trans person should not similarly be allowed to have their anatomy changed—without such evaluation or diagnosis—for seemingly much weightier reasons having to do with their very sense of self. In either case, it is sufficient for there to be good evidence that the intervention will likely benefit the individual in some way.

These considerations raise two likely objections that we wish to tackle before moving on to the next section. They concern distributive justice and the relevant account of well-being deployed here.

Distributive justice here relates to the allocation of health system funding and questions of priority. We have argued that we ought to conceptually decouple pathology from eligibility for a medical intervention. But pathology, to the extent that it corresponds to disadvantage or need, can be an indicator for how badly someone’s life will have gone if not helped (Kamm 2002). When resources are limited, society should prioritize those people whose lives, if they are not helped, will expectably go much worse than the life of someone whose interests would be set back only a little by a similar lack of help. Given this constraint, it follows that, regardless of the nature or root of a person’s gender dysphoria, those with a heightened experience of it are more greatly disadvantaged or may have a greater need to access certain gender affirming services, than those with mild or no dysphoria.

For that reason, we think a principle of remediating disadvantage (Persad 2021) will be key to guiding priority here. The worse off a beneficiary would be if not aided, the more important it is to benefit them all else being equal. However, it is crucial to emphasize that the welfarist approach is compatible with other accounts of justice as well: it is not in itself an argument for how to distribute scarce healthcare resources. Rather, it is an account that can help us think more clearly about what is actually at stake for individuals wishing to access transgender healthcare (i.e., the likely impact of various interventions on their well-being, rather than whether they have a specific pathology).

This brings us to the second objection that will likely be raised about our account. This is the concern that the welfarist account merely replaces one set of messy and unhelpful concepts—like disorder and normalcy—with an equally messy and unhelpful one: a better life.^3^

We have been referring to the notion of a better life synonymously with a life with more well-being. However, even if one agrees that a better life can be understood this way, there is no consensus about what constitutes well-being, so how do we determine whether someone accessing gender affirming services is likely to be enhanced and not further disadvantaged?

We do not think this objection is very compelling. Firstly, some interventions are clearly enhancing on any plausible conception of a good life for most people in ordinary circumstances. This is the case for the majority of interventions that aim to restore health or prevent disease. However, even when there is disagreement, patient preference and their own conception of the good life is central to any philosophy of medicine and medical decision-making that is not highly paternalistic.

Consider a patient wishing to undergo a vasectomy: a physician and patient may discuss the procedure’s effectiveness and benefits, but also its risks, such as postvasectomy pain syndrome. However, none of this would be relevant unless the patient first has a clear preference for being sterilized: their preference not to risk pregnancy is key to this deliberative process. Also relevant are the person’s particular circumstances: perhaps they have a partner who does not tolerate the contraceptive pill, or perhaps they or their partner are allergic to latex or simply dislike condoms. This is where the welfarist account’s reference to the “relevant set of circumstances” kicks in: medicine is context-dependent and any conception of a good life will be tied to an individual’s circumstances, whether personal, social, political, or otherwise. It is the patient’s conception of a good life—with reference to uncontroversial values such as friendship and autonomy—guided by the physician’s expertise on the benefits and risks of an intervention, that characterizes this ideal of participative decision-making between physician and patient (Synofzik 2009). (Which is to say, this is not merely a desire-satisfaction account of well-being.)^4^

Ultimately, in cases of gender dysphoria, it is uncontroversial to believe that distress, especially significant ongoing distress, is a marker of ill-being and that alleviating distress improves well-being. The question is what particular bodily or mental changes will most likely promote well-being and what will likely *most* promote it given the circumstances of a particular individual (given relevant alternatives).

## Controversial Cases of Gender Affirmation

In this final section we move on to the remaining controversies associated with the diagnostic approach previously highlighted.

### The Presence of Psychological Disorders

As noted, some individuals with gender dysphoria may also suffer from other underlying psychological disorders or, in the welfarist sense, psychological conditions or dispositions that are typically disadvantageous. There is some evidence that trans individuals with autism spectrum disorder may be blocked from accessing gender affirming services (White 2016), and more generally there may be a concern that in some (even if very few) cases the wish to transition is itself symptomatic of autistic or other atypical traits. This concern may be expressed as one about the likely success of various medical interventions at reducing gender dysphoria, since the interventions would presumably not be responding to the “real” underlying issue.

The welfarist account provides a framework for thinking about these cases. Firstly, whether autism is a medical condition on a naturalistic account of disease with identifiable biomarkers is largely irrelevant here—indeed, as we noted previously, some have argued it should not be considered a disorder (O’Neil 2008). As with gender dysphoria, the welfarist account frames autism as a potentially disadvantageous bundle of psychological dispositions, with the degree of disadvantage being determined by the impact on the individual’s overall well-being in their given context.

Rather than getting side-tracked by trying to determine which is the “real” or (most) underlying disorder, the central question for the welfarist is which response to the individual is most likely to improve their well-being given the presence of autistic traits. Even if the wish to affirm a particular gender identity *is* something that arises in large part from having autistic traits, in some instances it may still be the case that responding to that wish by ameliorating the distress through body modification will improve overall well-being. On the other hand, it is also relevant to ask whether attempting to address or mitigate some of the individual’s autistic (or other) traits could improve well-being *more*.

However, these are not questions that can be answered by arm-chair reasoning. Rather, they require serious empirical legwork. For instance, it would be relevant to take into account what limited data there is about the well-being impact of such interventions on individuals in similar circumstances who have already undergone them. While a majority of individuals who undergo so-called sex reassignment surgery note marked improvements in well-being, some studies suggest that about 20 per cent do not experience significant benefit (Murad et al. 2010) and some 2 per cent come to regret it (Dhejne et al. 2014)—although some have argued these estimates are likely conservative (Zucker, Lawrence, and Kreukels 2016). In the case of an individual with gender dysphoria and autistic traits, does the presence of the latter increase or decrease those chances of regret? Being able to answer these questions will be key to any meaningful informed consent arising from the deliberative process that characterizes—or ought to characterize—the patient-physician relationship.

### Prejudice

What about cases where a person’s distress or dysphoria is arising primarily from prejudice against one’s expressed gender identity or associated self-presentation rather than, say, an “internal” sense of misalignment or incongruence? To oversimplify somewhat, the diagnostic model looks to examine conditions internal to the body and mind, as opposed to distress that is contingent on or arising from prejudice or discrimination. If, therefore, one’s suffering is primarily rooted in external causes—such as sustained mistreatment by others—the diagnostic model may overlook such a person’s distress. It would be considered a social, economic, or political issue and therefore beyond the scope of medicine as it is traditionally conceived. (Needless to say, insofar as a person’s social context is oppressive or they are mistreated by others for any reason, those factors ought to be addressed *whether or not* the individual would be helped by access to medicine (Purdy 2001).)

In contrast, for the welfarist approach, the fact that someone is suffering because of society’s unjust response to their bodily or mental states does not necessarily mean their suffering is beyond the scope of medicine. It may be that, if there is no immediate, or sufficiently immediate, way to alter the society in which they live to mitigate that distress, changing their body or mind in a way that protects them from, say, society’s prejudice, would be enhancing and may be permissible (Earp 2014).

This is not as controversial as it sounds. Consider a person who is deaf and where the main source of their diminished well-being is society’s unjust failure to accommodate them. Suppose they are otherwise fully immersed in a deaf culture that they find fulfilling and rewarding but their chances of leading a good life are limited primarily due to the broader society of which they are a part. In such a case, their deafness may be disabling, not because the bodily state of being deaf is disabling in and of itself but because of a discriminatory society. Nevertheless, if this person decided that, all things considered, they still wanted to undergo a hypothetical intervention that allowed them to hear, this could be advantageous for them and in that sense enhancing (and prima facie permissible). Importantly, this is the case notwithstanding that that society has an obligation to better accommodate them as they are (for example, by making sign-language a mandatory part of general education (see Bowman-Smart et al. (2019)).

Similarly for some transgender individuals.^5^ It may be that in some cases their distress is at least partly due to society’s prejudiced response to their gender presentation: say, the fact that they dress or behave a certain way that does not align in the culturally prescribed manner with their real or perceived sex. One response to this would be to push for social change: indeed, in our view, society *should* change in significant ways, for example by becoming much more accepting of gender non-conforming people and behaviour. In the meantime, however, some transgender people may continue to be disadvantaged and—in the welfarist sense—disabled, by the ongoing non-ideal circumstances in which society is insufficiently accepting of their gender identity or presentation. Were they to undergo hormone treatments or sex reassignment surgery under such circumstances, this could still be all things considered beneficial for them, *even if* only as a way of avoiding prejudiced mistreatment as society changes. The welfarist account may label this an enhancement.

Of course, justice here is a further crucial consideration: the fact that something can be enhancing for an individual does not necessarily justify providing it. For instance, the fact that in some societies it is disadvantageous to be of a certain skin colour would not justify the provision of a service that alters skin colour—there may be good justice-based reasons to ban such a service and to instead enact laws against discrimination. A similar argument could be made about efforts to change sexual orientation (Delmas and Aas 2018; Earp, Sandberg, and Savulescu 2014b; Earp and Vierra 2018). However, unlike the suffering that many people with non-heterosexual orientations experience, gender dysphoria is not, as a rule, due solely to unjust social pressures. Instead, at least some significant proportion of transgender people seem to experience dysphoria for largely “internal” reasons, for example, reasons having to do with their psychobiological development (Case et al. 2017; Altinay and Anand 2020). Therefore, gender affirming technology should not be banned. Moreover, so long as it is available, anyone whose well-being would be on balance improved by the technology (including those whose distress *is* largely due to external factors) should have access to it, provided the resource constraint conditions outlined above are met.

### Cases Without Dysphoria

Finally, there may be trans individuals who experience neither “internal” dysphoria nor externally-driven distress but who wish to access gender affirming services nonetheless. Beischel, Gauvin, and van Anders (2021) and Ashley (2019) develop an account of “gender euphoria” and “creative transfiguration” as two reasons unrelated to dysphoria that may lead individuals to seek out hormone replacement therapy. No empirical data suggests there are very many such individuals, but it is plausible there could be more as the technology for transitioning becomes less invasive and costly. These would be cases that the diagnostic model, and the traditional account of medicine, completely neglect. The welfarist approach however can account for these individuals: if the use of gender affirming technologies is likely to improve a person’s well-being, despite any associated risks or harms, it may still count as an enhancement. To that extent, there is a prima facie reason to allow the person access (especially if, for example, the cost of the enhancement would be covered from private rather than public funds, in the way that other forms of enhancement, such as so-called cosmetic surgeries, are typically funded unless there is a perceived “medical” necessity for them).^6^ That is indeed how we think about cosmetic enhancements, which the welfarist account also accommodates.

## Conclusion

We have argued that the diagnostic model entails significant conceptual shortcomings that impede ethical deliberation when it comes to individuals seeking to access gender affirming services. Even after moving away from the label of “disorder,” the idea of receiving a diagnosis still entails the presence of a pathology or some medical condition that, in turn, warrants “treatment.” It may also make it more likely that a transgender identification will be seen as being rooted in a psychological disturbance which must then be the focus of treatment—e.g., in the case of individuals with autism who wish to affirm a particular gender. It also tends to neglect cases where the experience of distress is due to external factors like prejudice, as well as cases where there may be no distress at all.

In contrast, we have argued that the welfarist account of enhancement offers a much richer conceptual framework to think about the ethics of all these cases. The welfarist approach decouples medicine from pathology, gives equal weight to distress arising internally or externally, and can accommodate cases where there is no distress at all. As emphasized, the fact that a bodily or mental change may be an enhancement does not, on its own, settle questions about overall permissibility or justice. That gender affirmation may be enhancing for someone gives us a prima facie reason for them to access it, but that can in principle be outweighed by, for example, distributive justice considerations, at least in cases where public funds are used and the presenting individual is experiencing comparatively less distress.

## Footnotes

1 See Sholl (2017) for a distinction between the concept of medicalization as opposed to pathologization.

2 There is an extensive debate in the trans literature about the differences between transgender and so-called transracial changes to one’s body. We do not intend to wade into this debate and only raise the comparison here to highlight the potential tension between combating prejudice or discrimination and trying to evade it in one’s personal life by altering their body. See Heyes (2006) for a critique of the so-called “transracial analogy.”

3 For this objection, see Beck and Stroop (2015). For a response, see Zohny (2015).

4 No doubt people may be mistaken about their conception of a good life, or their conception of a good life may be incompatible with, for example, the demands of justice. These possibilities would need to be factored into any deliberative process preceding the undertaking of a medical intervention.

5 This argument is developed in more detail in chapters 2 and 11 of Earp and Savulescu (2020).

6 See Ashley and Ells (2018) for an argument for publicly funding certain trans surgeries even if they do not meet requirements associated with medical necessity.
